# Cytokinins Reduce Viral Replication and Alter Plaque Morphology of Frog Virus 3 In Vitro

**DOI:** 10.3390/v16060826

**Published:** 2024-05-23

**Authors:** Mark Seegobin, Samantha R. Logan, R. J. Neil Emery, Craig R. Brunetti

**Affiliations:** Department of Biology, Trent University, 1600 West Bank Drive, Peterborough, ON K9J 0G2, Canada; markseegobin@trentu.ca (M.S.); samanthalogan@trentu.ca (S.R.L.); nemery@trentu.ca (R.J.N.E.)

**Keywords:** ranavirus, frog virus 3, viral replication, cytokinins, phytohormones, antiviral agents, *Iridoviridae*

## Abstract

Cytokinins (CKs) are a group of N^6^-substituted signaling molecules whose biosynthesis and metabolism have been documented in all kingdoms of life, including vertebrates. While their biological relevance in vertebrate systems continues to be elucidated, they have broadly been documented with therapeutic effects in exogenous applications. In this study, we evaluated the virostatic potential of four types of CKs including, *N*^6^-isopentenyladenine (iP), *N*^6^-isopentenyladenosine (iPR), *N*^6^-isopentenyladenosine-5′monophosphate (iPMP), and 2-methylthiol-*N*^6^-isopentenyladenosine (2MeSiPR) against the ranavirus type species, frog virus 3 (FV3). Following concurrent treatment and infection, iP and iPR reduced viral replication by 33.8% and 59.6%, respectively, in plaque formation assays. A decrease in viral replication was also observed when CK exposure was limited to 12 h prior to infection, where iP and iPR reduced viral replication by 31% and 23.75%, respectively. Treatment with iP and iPR was also marked by 48% and 60% decreases in viral load over 72 h, respectively, as measured in single step growth curves. Plaque morphology was altered in vitro, as iP and iPR treatment increased plaque area by 83% and 112% with lytic zone formation also becoming more prevalent in corresponding treatments. Treatment with iPMP and 2MeSiPR resulted in no effect on viral kinetics in vitro. The results of this study are the first to provide evidence of CK antiviral activity against a DNA virus and highlight the importance of their structure for therapeutic investigations.

## 1. Introduction

**Figure 1:**
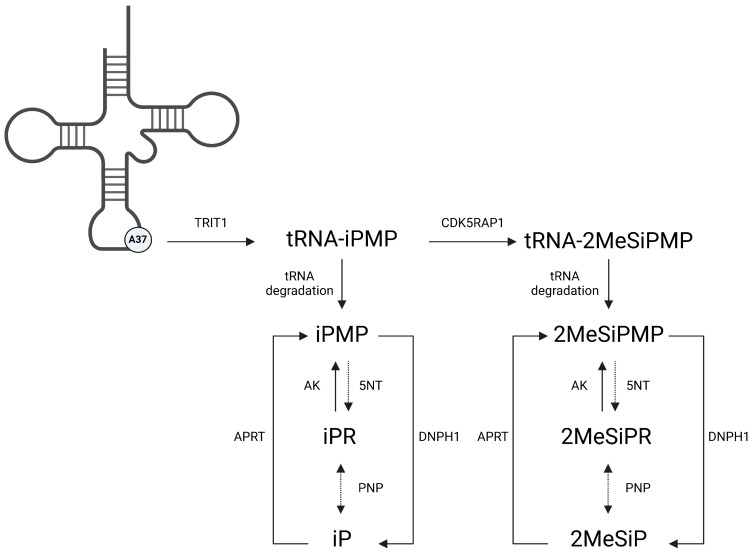
Cytokinin biosynthesis and metabolism via the mevalonate pathway in vertebrate systems. In a subset of tRNAs, A37 is modified by tRNA-isopentenyltransferase, TRIT1, producing tRNA-*N*^6^-isopentenyladenosine 5′ monophosphate (tRNA-iPMP; i^6^A37). Subsequent modification by tRNA-methylthiotransferase, CDK5RAP1, produces tRNA-2-methylthiol-*N*^6^-isopentenyladenosine 5′ monophosphate (tRNA-2MeSiPMP; ms^2^i^6^A37). Following tRNA degradation or exogenous application, these compounds are metabolized via the purine salvage pathway through which they may exert their biological and therapeutic effects. 5NT, 5′ nucleotidase; AK, adenosine kinase, PNP; purine nucleoside phosphorylase; APRT, adenine phosphorylribosyltransferase; DNPH1, 2′ deoxynucleoside 5′-phosphate N-hydrolase 1. Created with BioRender.com.

Cytokinins (CKs) are a group of *N*^6^-substituted adenylate derivatives most widely known as plant signaling molecules. Although traditionally considered phytohormones, CKs are not exclusive to plant systems with CK biosynthesis genes being documented in all kingdoms of life [1,2,3,4]. Two vertebrate CK biosynthesis genes, *trit1* [5] and *cdk5rap1* [6,7], modify A37 on a subset of tRNAs to produce tRNA-*N*^6^-isopentenyladenosine 5′-monophosphate (tRNA-iPMP; i^6^A37) and tRNA-2-methylthio-*N*^6^-isopentenyladenosine 5′-monophosphate (tRNA-2MeSiPMP; m^2^i^6^A37). During tRNA degradation these adenine derivatives are released and metabolized via purine salvage pathways (Figure 1). In vertebrates, reports outlining endogenous detection remain relatively scarce although tRNA-free CKs have been detected both in vivo and in vitro [8,9].

For example, among 21 primary canine tissues, mass spectrometric scanning detected seven types of CKs (ranging in concentration from 1.96 to 1.40 × 10^3^ pmol/g fresh weight) including: *N*^6^-isopentenyladenine (iP), *N*^6^-isopentenyladenosine (iPR), *N*^6^-isopentenyladenosine-5′ (mono-, di-, or tri-) phosphate (iPRP), 2-methylthiol-*N*^6^-isopentenyladenine (2MeSiP), 2-methylthiol-*N*^6^-isopentenyladenosine (2MeSiPR), and 2-methylthiol-zeatin (2MeSZ) [9]. Likewise, this suite of CKs was detected in HeLa cell culture (intracellular, ~0.02–0.34 pmol/10^6^ cells; extracellular, 0.03–2.17 nM) [8] and among culture medias used for culturing vertebrate cell lines (~3.00–43.48 pmol/5 mL) [10]. Although the presence, uptake, and interconversion of tRNA-free CKs has been documented in vertebrate tissues and cells, their role remains elusive [8,9,10,11]. Whereas there are few studies highlighting endogenous CK detection, there are numerous reports outlining the therapeutic potential of CKs in exogenous applications. In particular, CK nucleosides have been characterized with pleiotropic biological effects, including anti-tumor and anti-angiogenic activity both in vitro and in vivo at concentrations ranging from 1 to 25 μM [12,13,14,15,16,17,18,19,20,21,22,23]. Anticancer activity of CKs has been linked to DNA-binding [12], ATP depletion [13,14,15], AMPK activation [11,16,17], Akt/NF-kB pathway suppression [18,19], JNK phosphorylation [20], oncogenic protein prenylation inhibition [17,21] and NK cell regulation [22,23].

Given the evidence for therapeutic potential and elucidation of CK-mediated cell responses, it is surprising how few studies have evaluated their antiviral activity [24,25,26]. In screening campaigns against RNA viruses, the aromatic cytokinin *N*^6^-benzyladenosine (BAR) was identified as an active inhibitor of both Marburg [24] and Lassa viruses [25]. Recently, another aromatic cytokinin, kinetin, was identified as an inhibitor of SARS-CoV-2 replication; reducing the viral induction of IL-6 and TNF levels as well as inducing error-prone vRNA synthesis [27]. Additionally, iPR was effective at reducing human enterovirus 71 strain BrCr (EC_50_ = 1.0 ± 0.2 µM) and several clinical isolates (EC_50_ = 1.2 ± 0.2 to 2.5 ± 0.5 µM) in vitro [26]. The mechanisms behind this antiviral activity remain elusive; however, it has been proposed that CKs may impede viral entry or interact with host machinery involved in translation [26,28]. While the few existing studies have evaluated CK activity against RNA viruses, to date there have been no studies examining their efficacy when challenging DNA viruses, such as the ranavirus type species, frog virus 3.

Ranaviruses, of the family *Iridoviridae,* comprise a group of emerging large double- stranded DNA viruses infectious to cold-blooded vertebrates including amphibians, reptiles, and fish [29]. The ranavirus type species, frog virus 3 (FV3), is the best characterized at the molecular level and has served as the model species amongst ranaviral research [30]. Previous FV3 studies have characterized features applicable across all *Iridoviridae,* including temporal transcription of coding genes, phage-like hyper-methylated genomic DNA, virus-mediated arrest of host immune responses, and two-stage viral genome replication [29,31]. These advances have also helped delineate the evolution and host tropism diversity among iridoviruses and other nucleocytoplasmic large DNA viruses (NCLDV), providing greater resolution to the evolutionary history of viruses associated with different host systems [32,33].

Given the impact of previous FV3 studies including the characterization of viral replication and infection, along with emerging analyses of the viral genome and transcriptome, FV3 is an excellent model for exploring candidate antiviral agents. Thus, in our study we are the first to examine the antiviral potential of four isoprenoid CKs (iP, iPR, iPMP, and 2MeSiPR) in DNA viruses. Here, we report that both iP and iPR significantly reduce FV3 replication and increase plaque size, suggesting an enhancement in viral spread from cell to cell.

## 2. Materials and Methods

### 2.1. Cytokinin Solution Preparation

Stock N^6^-(Δ^2^-isopentenyl) adenine (iP), N^6^-(Δ^2^-isopentenyl) adenine-9-riboside (iPR), N^6^-(Δ^2^-isopentenyl) adenine-9-riboside-5′-monophosphate (iPMP) and 2-methylthio-N^6^-isopentenyladenosine (2MeSiPR) (OlChemIm Ltd., Olomouc, Czech Republic) solutions were prepared at 10 mM in DMSO with DMSO not exceeding 0.5% in media.

### 2.2. Epithelioma Papulosum Cyprini (EPC) Cells

Epithelioma papulosum cyprini cells (EPC, American Type Culture Collection, ATCC No. CRL-2872) were maintained in non-vented culture flasks containing Leibovitz’s L-15 media (L-15; Thermo Fisher Scientific, Waltham, MA, USA) containing 2.0 mM L-glutamine supplemented with 10% fetal bovine serum (FBS), 100 U/mL penicillin, 100 μg/mL of streptomycin, and 1.5 μg/mL of amphotericin B (Thermo Fisher Scientific, Waltham, MA, USA) at 20–25 °C. Every 5 to 7 days, EPC cells were sub-cultured 1:8 by rinsing with phosphate buffered saline (PBS, pH 7.2, Thermo Fisher Scientific, Waltham, MA, USA) and treatment with TrypLE (Thermo Fisher Scientific, Waltham, MA, USA) to detach the adherent cells.

### 2.3. FV3 Propagation in EPC Cells

FV3 (ATCC No. VR-567) was propagated on confluent monolayers of EPC cells in 75 cm^2^ flasks at a multiplicity of infection (MOI) of 0.1 PFU/cell. Infected EPCs were incubated at 20–25 °C and harvested 5 days post-infection when approximately 90% of the monolayer displayed cytopathic effects. Virus stocks were freeze-thawed in three cycles to promote cell lysis and were partially purified by centrifugation (4000 RPM, 5 min). Virus stocks were stored at −80 °C and titers were determined by standardized plaque assay.

### 2.4. Plaque Formation and Morphology Assays

In 6-well plates, 1.7 × 10^6^ EPC cells were seeded using L-15 media supplemented with 10% FBS to form confluent monolayers. EPC cells were concurrently infected with FV3 at an MOI of 0.02 PFU/cell in L-15 supplemented with 1% FBS and iP, iPR, iPMP or 2MeSiPR (0–20 μM) for 24 h. Controls were incubated in L-15 supplemented with 1% FBS and an equal amount of solvent (0.5% *v*/*v* DMSO). To evaluate the treatment timing, CKs were also applied 12 h before infection after which the supernatant was removed, the cells were washed three times with PBS (Thermo Fisher Scientific, Waltham, MA, USA), and then infected with FV3. Following treatment and infection, supernatants were removed, monolayers were washed three times with PBS and overlaid with 0.75% methylcellulose (Sigma-Aldrich, Oakville, ON, Canada) in L-15 supplemented with 1% FBS. Seventy-two hours post infection, plaque formation and morphology were assessed. Plaques were counted using a Nikon Ts2R-FL inverted microscope (Nikon Canada Incorporated Instruments Division, Mississauga, ON, Canada) and plaque formation was calculated relative to the DMSO control. To evaluate morphology, plaques were imaged using an EVOS XL Auto Imaging System (Thermo Fisher Scientific, Waltham, MA, USA) and ImageJ v1.53k [34] was used to calculate plaque area.

### 2.5. Single-Step Growth Curves 

In 6-well plates, EPC cells were prepared as previously described for plaque formation assays to form confluent monolayers. Cultures were concurrently infected with FV3 at an MOI of 5 and treated with 16.5 μM iP or 16 μM iPR in L-15 supplemented with 1% FBS to achieve 75% maximum inhibition of viral replication based on initial plaque formation assays (Figure S1). After 4 hours at room temperature, supernatants were removed, monolayers were washed three times with 1x PBS (pH 7.2), and media was replaced with L-15 supplemented with 1% FBS and the appropriate concentration of the corresponding compound. Samples were collected at various time points over 72 h and virus titers were determined by plaque assay.

### 2.6. Cytotoxicity and Proliferation Assays 

The cytotoxicity and proliferative effects of all compounds were evaluated using a Cell Counting Kit 8 assay (WST-8/CCK8; Abcam, Toronto, ON, Canada) in 96-well plates seeded with 2 × 10^5^ cells and 1 × 10^5^ cells/well, respectively. Cells were treated with iP, iPR, iPMP or 2MeSiPR (30 μM) in 1% L-15 for 24 h at 20–25 °C. Regarding proliferation assays, after the initial 24 h exposure supernatants were replaced with fresh 1% L-15 and plates were incubated for an additional 48 h. Subsequently, 10 μL of CCK-8 solution was added to each well and plates were incubated for 3 hours. Absorbance was measured at OD 460 nm using a microplate reader (SpectraMax M3, Molecular Devices, San Jose, CA, USA). Cytotoxicity and proliferation assays were performed twice, comprising three replicates each time.

### 2.7. Statistical Analysis

Data distribution was first evaluated using a Shapiro–Wilk test in GraphPad Prism 8 (GraphPad Software Incorporated, La Jolla, CA, USA). Outcomes following a normal distribution were evaluated using a one-way ANOVA followed by a Tukey’s test or a two-way ANOVA followed by a Bonferroni multiple comparison. Otherwise, data were evaluated using a Kruskal–Wallis test followed by Dunn’s post hoc analysis. A *p*-value < 0.05 was considered significant and *n* represents the number of replicates from independent experiments.

## 3. Results

### 3.1. iP and iPR Reduce Relative Plaque Formation during FV3 Infection

**Figure 2:**
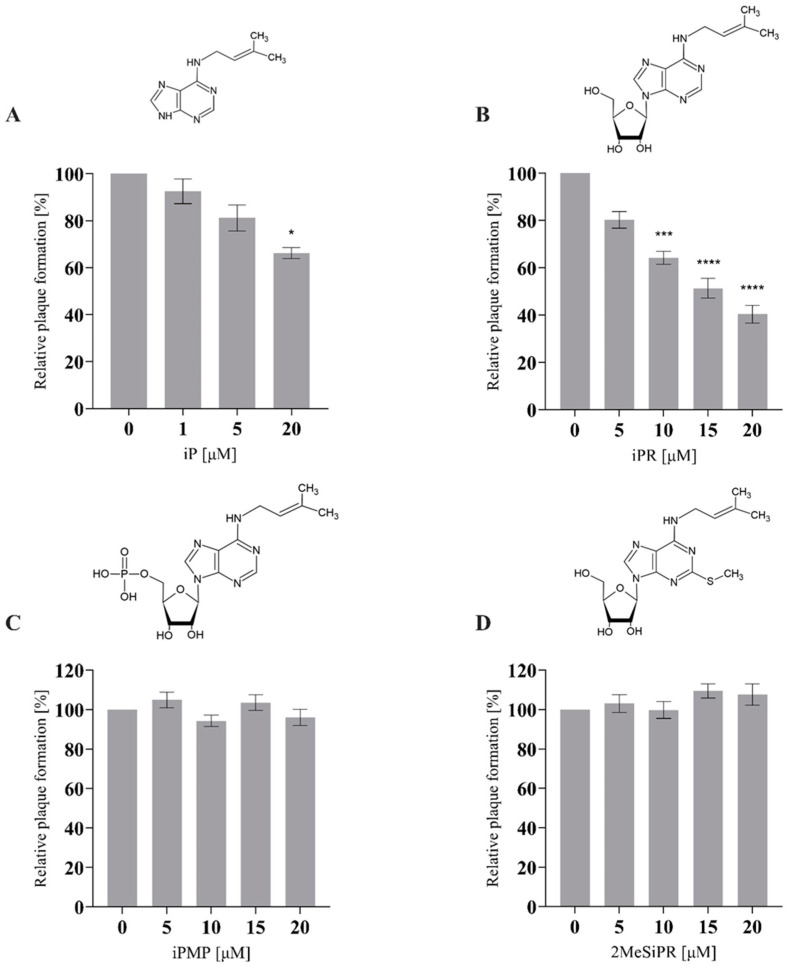
iP and iPR reduce plaque formation during FV3 infection. EPC cells were concurrently infected with FV3 and treated with exogenous (**A**) iP, (**B**) iPR, (**C**) iPMP, or (**D**) 2MeSiPR at 0–20 μM for 24 h. After 24 h, cells were overlaid with 0.75% methylcellulose in L-15 media supplemented with 1% FBS. Plaque formation was assessed 72 h post-infection. Data are presented as mean relative plaque formation (% of control) ± SEM. Statistical significance was evaluated using a Kruskal–Wallis test followed by Dunn’s post hoc analysis (n ≥ 3; * *p* ≤ 0.05, *** *p* ≤ 0.001, **** *p* ≤ 0.0001).

The effects of exogenous cytokinin application (0–20 µM) during FV3 replication were assessed by plaque formation assay (Figure 2). iP and iPR significantly reduced the number of plaques formed relative to controls during FV3 infection. iP significantly reduced plaque formation by 33.8% (*p* = 0.0315) at 20 µM. iPR significantly reduced plaque formation by 36.8% (*p* = 0.0005), 48.6% (*p* < 0.0001) 59.6% (*p* < 0.0001) at 10, 15, and 20 µM, respectively. No significant changes in relative plaque formation were observed when iPMP or 2MeSiPR were applied during infection (Figure 2).

**Figure 3:**
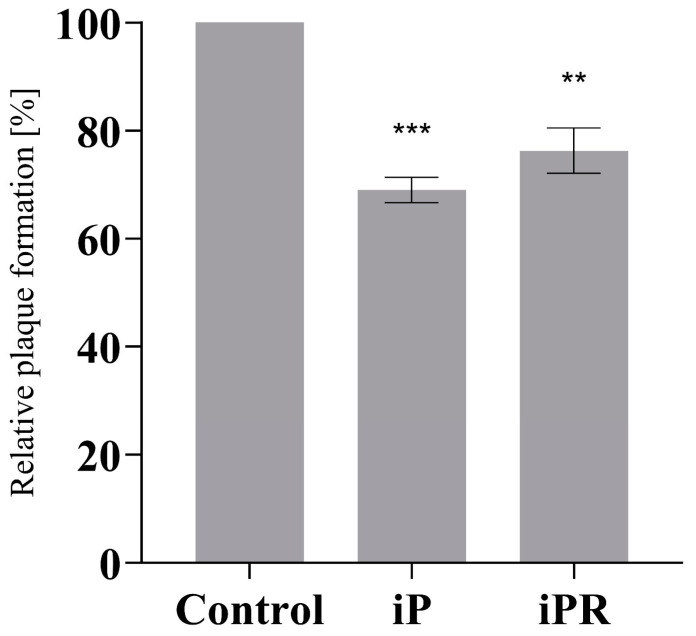
Pretreatment with iP and iPR reduce plaque formation during FV3 infection. EPC cells were treated with exogenous iP and iPR at 20 μM for 12 h. The iP or iPR was removed and cells were infected with FV3 for 24 h. After 24 h, cells were overlaid with 0.75% methylcellulose in L-15 media supplemented with 1% FBS. Plaque formation was assessed 72 h post-infection. Data are presented as mean relative plaque formation (% of control) ± SEM. Statistical significance was evaluated using a Kruskal–Wallis test followed by Dunn’s post hoc analysis (n = 3; ** *p* ≤ 0.01, *** *p* ≤ 0.001).

Additionally, 20 µM of iP or iPR were applied 12 h prior to infection to assess the importance of treatment timing (Figure 3). Following cytokinin treatment, the supernatant was removed, EPC cells were washed with PBS, and then infected with FV3 at an MOI of 0.02 PFU/mL for 24 h. As observed in concurrent treatments, plaque formation was significantly reduced by pre-treatment by 31% (*p* = 0.002) and 23.75% (*p* = 0.0026) for iP and iPR, respectively. Furthermore, no cytotoxicity or changes in proliferation were observed for these compounds as determined using cell viability assays (Table S1).

### 3.2. iP and iPR Begin to Significantly Reduce FV3 Titers after 30- and 16-h Post Infection, Respectively

**Figure 4:**
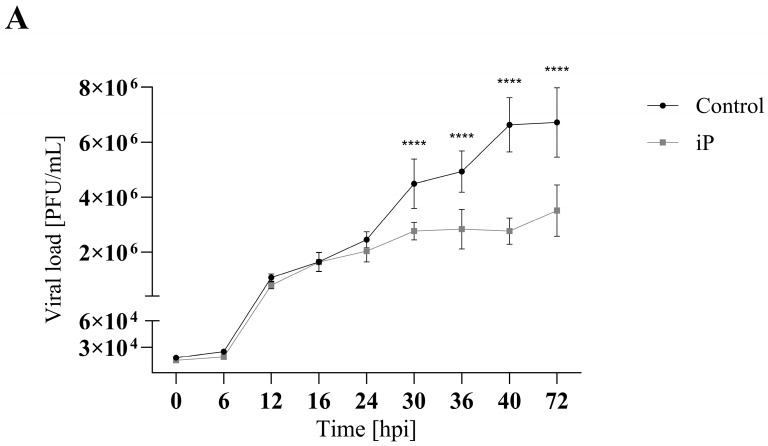

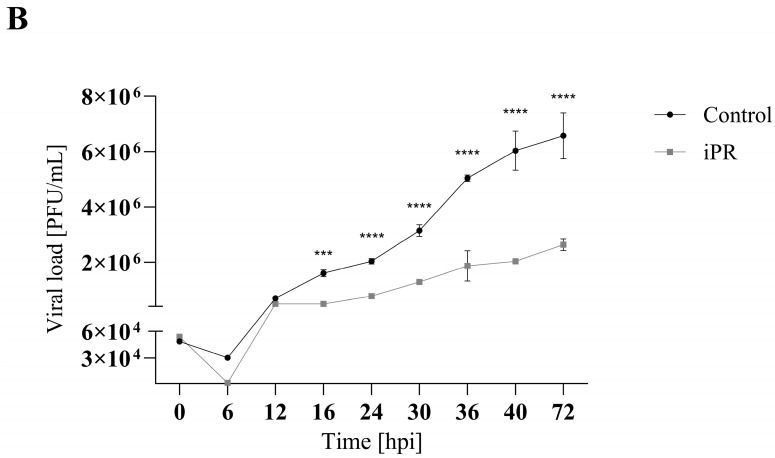
iP and iPR begin to reduce FV3 titers at 30 and 16 h post infection, respectively. Equivalent numbers of EPC cells were simultaneously infected with FV3 at an MOI of 5 and either (**A**) 16.5 μM of iP or (**B**) 16 μM of iPR. The virus was harvested at the indicated time points and quantified in triplicate by plaque assay. Data are presented as mean PFU/mL ± SD. Statistical significance was evaluated using a two-way ANOVA followed by a Bonferroni post hoc test (n = 1; *** *p* ≤ 0.001, **** *p* ≤ 0.0001).

To evaluate the effect of cytokinins on viral replication over time, a single-step growth curve was performed for FV3 (MOI 5) in EPC cells over a 72 h period with and without CK treatment. EPC cultures were treated with cytokinin to achieve 75% of the maximum reduction in viral replication as determined by plaque formation assay; 16.5 µM iP or 16 µM iPR (Figure 4 and Figure S1). Infections treated with iP exhibited similar viral replication kinetics to control groups over the first 24 h post infection (h.p.i.) after which they began to diverge. Viral titers significantly decreased in iP-treated infections after 30 h.p.i. by 38% (*p* < 0.0001) leading to a 48% (*p* < 0.0001) reduction in viral load by 72 h.p.i. Similarly, infections treated with iPR were initially comparable to controls for 12 h.p.i.; however, treated cultures diverged earlier as viral titers significantly decreased after 16 h.p.i. by 69% (*p* = 0.0006), leading to a 60% (*p* < 0.0001) reduction in viral load by 72 h.p.i.

### 3.3. iP and iPR Alter FV3 Plaque Size and Morphology

**Figure 5:**
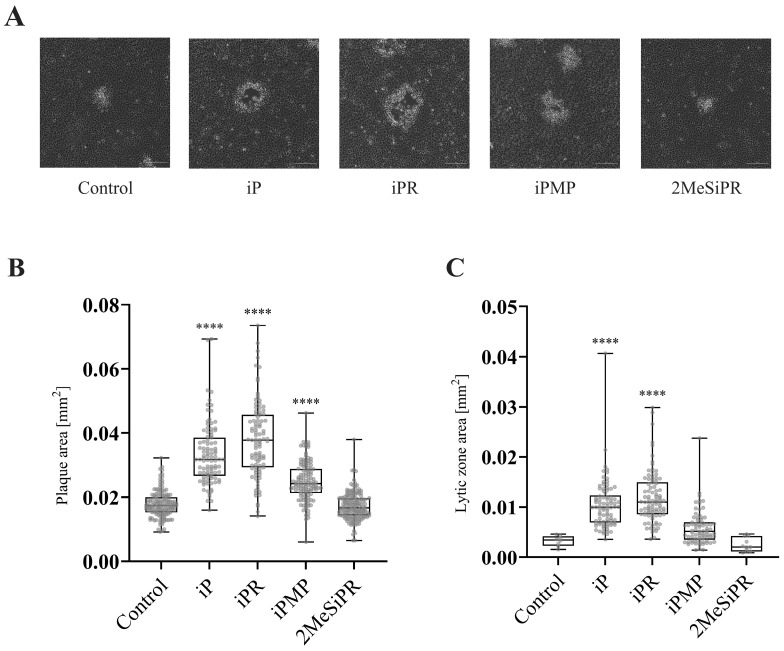

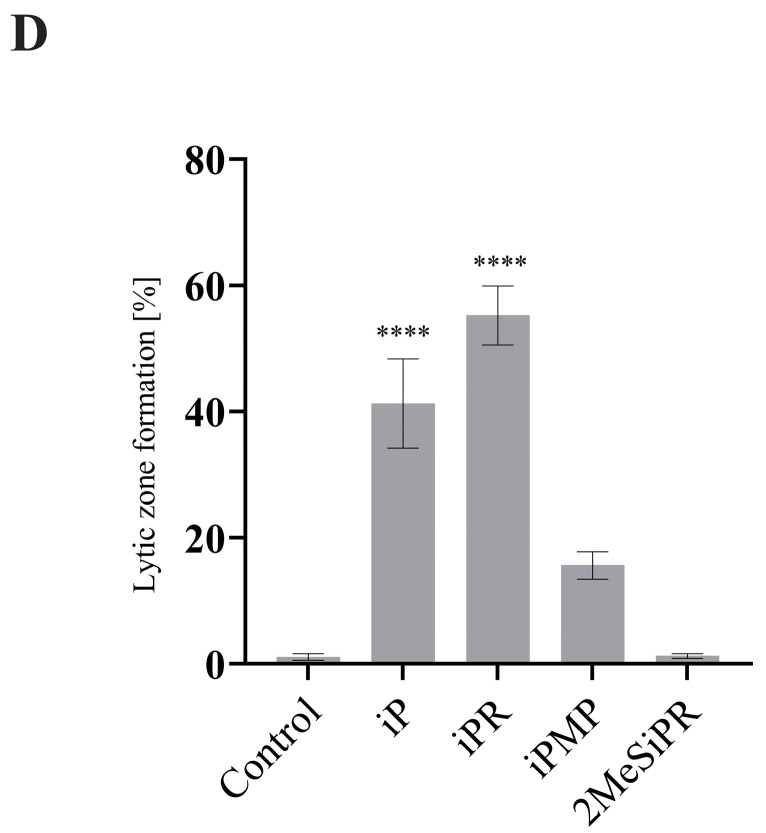
iP and iPR alter FV3 plaque area and morphology. (**A**) EPC cells were simultaneously infected with FV3 and treated with 20 μM of iP, iPR, iPMP, or 2MeSiPR for 4 h. Subsequently, inocula were removed and replaced with 1% media containing 20 μM of the corresponding cytokinin. After 24 h, cells were overlaid with 0.75% methylcellulose supplemented with 1% FBS. Images were taken 72 h post infection. Scale bar = 50 μm. Images are representative of three independent experiments. (**B**) Total plaque area and (**C**) lytic zone area were quantified using ImageJ with data presented as plaque area (mm^2^). Statistical significance was assessed using a Kruskal–Wallis followed by Dunn’s post hoc analysis (n = 3; **** *p* ≤ 0.0001). (**D**) The relative presence of lytic zones for each group was also measured for context. Data are presented as mean lytic zone formation (% of total) ± SEM. Statistical significance was assessed using a one-way ANOVA followed by a Tukey’s multiple comparisons test (n = 3; **** *p* ≤0.0001).

Since viral production was reduced, we wanted to determine if plaque morphology was also affected (Figure 5). To quantitatively assess plaque size and lytic zone formation, EPC cells were infected with FV3 and plaque area was assessed after 72 h.p.i. using ImageJ (Abramoff et al., 2004). Total plaque area significantly increased by 83% (*p* < 0.0001), 112% (*p* < 0.0001), and 40% (*p* < 0.0001) when EPC cells were treated with 20 µM iP, iPR, or iPMP, respectively. No significant change in plaque area was observed following 2MeSiPR treatment (Figure 5). Lytic zone formation was also more prevalent in monolayers treated with iP and iPR. A significant number of plaques exhibited lytic zone formation in both iP (41%, *p* < 0.0001) and iPR (55%, *p* < 0.0001) treated groups (Figure 5).

## 4. Discussion

While the therapeutic application of cytokinins (CKs) has primarily been targeted towards cancer treatments, here we expand on their potential by exploring their antiviral properties in the ranavirus type-species, FV3. In this study, concurrent and pretreatment with N^6^-(Δ^2^-isopentenyl) adenine (iP) and N^6^-(Δ^2^-isopentenyl) adenosine (iPR) significantly reduced FV3 replication (Figure 2, Figure 3 and Figure 4). Additionally, iP, iPR, and N^6^-(Δ^2^-isopentenyl) adenosine-5′-monophophate (iPMP) altered plaque morphology (Figure 5), while 2-methylthio-N^6^-isopentenyladenosine (2MeSiPR) elicited no impact (Figure 2, Figure 4 and Figure 5).

In this study, of the four cytokinins evaluated only the iP type freebase and riboside demonstrated antiviral activity. Our findings are consistent with reports outlining a reduction in viral replication observed in RNA virus infections following treatment with CK ribosides [24,25,26,27]. In most cases, the underlying mechanisms have yet to be characterized; however, it has been suggested that CK activity may impede viral entry or limit translation [26]. It has also been suggested that conversion of CK into their nucleotide form is required for their activation [27]. In SARS-CoV-2 infected Calu-3 cells, kinetin and its corresponding nucleotides impair SARS-CoV-2 replication via inhibition of viral polymerase and incorporation into viral RNA [27]. While we did not evaluate the effect of CK treatment on host translational machinery, FV3 entry did not seem to be impacted based on our single-step growth curve in which viral replication was not significantly reduced until 16–30 h post infection (Figure 4). While our single-step growth curves were carried out at room temperature, evaluation of viral adsorption and entry can be improved by implementing temperature-mediated synchronous infection [35]. Additionally, viral replication was also significantly reduced in cultures pre-treated with iP and iPR up to 12 h prior to infection demonstrating the ability to prime for an antiviral response (Figure 3). Considering that CK interconversion and metabolism have been observed up to 72 h following exogenous application in vitro [8,36], further evaluation of both pre- and post- infection applications should be conducted to establish an effective treatment window.

Interestingly, a significant increase in plaque area was observed in iP, iPR, and iPMP-treated infections, as well as an earlier emergence of a lytic zone in iP and iPR-treated infections relative to controls (Figure 5). Typically, larger plaque size is correlated with greater fitness, which is associated with increased viral replication leading to earlier apoptosis and viral egress [37]. Despite a decrease in viral replication as indicated by reduced plaque formation (Figure 2) and viral load (Figure 4), the rapid shift in plaque morphology may suggest an earlier induction of apoptosis and cell-to-cell spread following CK treatment. No cytotoxic activity was observed in EPC cultures treated with CKs via viability assays; however, CK activation of apoptotic pathways is commonly observed in cultures derived from a range of cancers [36,38]. This suggests that CK induction of apoptotic pathways may in fact be limited to conditions of stress and inflammation, both of which are common to cancer and viral infection. The specific mechanisms of CK-attenuated virus replication remain to be determined; however, it is also possible that CK treatment interferes with viral evasion of the host immune system, leading to earlier apoptosis. Future research should more closely evaluate cell-to-cell spread and expression of apoptotic pathways following CK treatment during infections.

Ranavirus infection elicits a myriad of effects on host functions including inhibition of host macromolecule synthesis and a robust inflammatory response [29]. In response to viral infection, protein kinase R (PKR) is activated to inhibit translation via phosphorylation of eIF-2α; however, due to the synthesis of large amounts of highly efficient viral transcripts and FV3-encoded vIF-2α, a pseudo substrate, viral protein synthesis persists [39,40,41]. Recently, it was reported that iPR supplementation (0–4 mM) to culture media leads to incorporation in cellular RNA during transcription, resulting in a global reduction of protein synthesis, potentially compromised protein quality as measured by ER stress, and a decrease in translational fidelity as measured by protein aggregation [42]. Additionally, incorporation of iPR into RNA may allow for targeted cleavage by DNAzymes as an alternative mechanism to inhibit viral protein synthesis [43]. Integration of CKs into the viral genome has previously been highlighted as a factor in their virostatic potential [27]. The antiviral activity of kinetin has been linked to its integration into viral RNA where it is predicted to increase the width of double-stranded RNA and introduce non-Watson–Crick interactions which likely lead to error-prone SARS-CoV-2 replication [27]. Thus, it is plausible that viral genome replication and protein synthesis are limited following iPR treatment.

Ranaviral infections are associated with the induction of host immune signals such as interferon (IFN) and pro-inflammatory responses including TNF-α, IL-1β, IL-8, IL-17C, and IL-12 [44,45]. FV3 ORFs have been implicated in the evasion of these immune responses including a viral homolog of the tumor necrosis factor receptor (vTNFR) to intercept TNF-α signaling and DNA methyltransferase to prevent induction of IFN [46,47]. CKs may attenuate these viral evasion strategies as they can stimulate both pro- and anti-inflammatory immune responses. Kinetin significantly reduces viral induction of IL-6 and TNF-α in Calu-3 cells infected with SARS-CoV-2 [27]. In natural killer cells, iPR treatment stimulates pro-inflammatory immune responses including upregulation of IFN-γ, TNF-α, and CCL3/CCL5 chemokines [22,23]. Anti-inflammatory responses to iPR treatment have been documented in Chinese hamster ovary (CHO) cells where they are mediated through binding to adenosine A_3_ receptor [48,49] and in cystic fibrosis cell lines via inhibition of NFκB and STAT3 pathways [50]. Cytokinins are also known for their ability to stimulate AMPK activation as iPR is converted to iPMP via adenosine kinase (ADK) intracellularly (Figure 1). AMPK is a key regulator responsible for maintaining energy levels within a cell, active during times of stress to generate energy through catabolic pathways while simultaneously inhibiting anabolic processes [51]. While AMPK activation has not been evaluated during FV3 infections, CK activation of AMPK can lead to DNA damage and induction of apoptosis via caspases 3 and 9 [11]. In future work, AMPK activation should be characterized during FV3 infection to determine if it is utilized as part of the viral replication strategy.

While iP and iPR transport and receptor recognition have been documented in vitro [16,49,52], there have been no reports to characterize these interactions for CK nucleotides or 2MeS-types in vertebrate systems. The limited response to iPMP treatment may be due to its capacity to enter the cell or elicit a signal from the extracellular space. Before transport into the cell, extracellular AMP must be converted into adenosine via CD79, an ecto-5′-nucleotidases [53]. While CD79 has been reported to have weaker affinity to 5′-nucleoside monophosphates other than AMP (i.e., CMP, TMP, GMP, UMP and IMP), other substrates including ADP, ATP, and modified monophosphates with additional N^6^ side chains have demonstrated inhibitory activity [54]. Interactions between iPMP and vertebrate ecto-5′-nucleotidases have yet to be characterized; however, it is possible that substrate affinity for CD79 is limited, or inhibitory activity is preventing the conversion from iPMP to iPR in the extracellular space. No activity was observed for 2MeSiPR in our study. Despite endogenous detection of 2MeS CKs in vertebrate systems, very little is known about its role beyond its function as modifications in tRNA [9]. While 2MeS transport may be restricted in a similar manner as we have suggested for iPMP, the addition of the methylthiol group may prevent intracellular activity as observed for the iP-species. The limited response to iPMP and 2MeSiPR highlights the importance of CK riboside and free base structures to inhibit FV3 replication.

In this study, we describe the response of FV3 to the exogenous application of a family of adenine-derivative signaling molecules called cytokinins. Of the four CKs we examined, the application of iP and iPR significantly reduced FV3 replication while altering plaque morphology. This is the first study to report on CK antiviral activity against vertebrate DNA viruses, and while further work is required to determine their mechanism of action, our findings contribute to the characterization of endogenous CKs and expand upon their virostatic potential.

## Data Availability

Data are contained within the article or Supplementary Materials.

## Supplementary Materials

The following supporting information can be downloaded at: https://www.mdpi.com/article/10.3390/v16060826/s1, Figure S1: Regression analysis of plaque reduction following iP and iPR treatment to determine optimal concentration ranges for plaque formation assays and single-step growth curves; Table S1: The cytotoxic (CC_50_) and proliferative (EC_50_) concentrations of cytokinins in EPC cells after 24- and 72-h exposure, respectively.
