# Supplementary material for: Cytokinins Reduce Viral Replication and Alter Plaque Morphology of Frog Virus 3 In Vitro
Source: Viruses. 2024 May 23;16(6):826. doi: 10.3390/v16060826 (PMC11209418; doi:10.3390/v16060826)
Supplement: Supplementary file 1 [file viruses-16-00826-s001.zip › Figure S1.pdf]

A

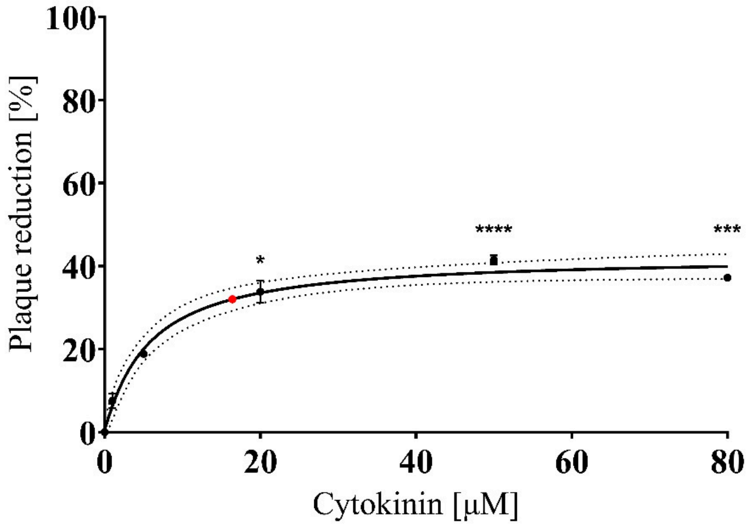

B

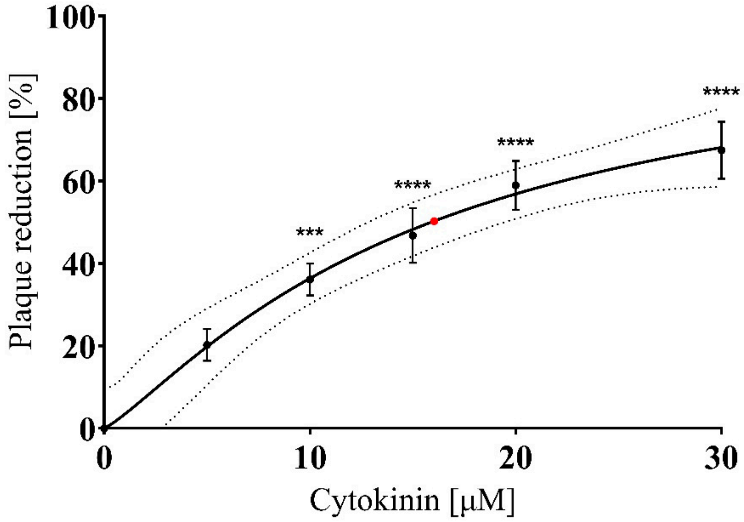

Supplementary Figure S1. Regression analysis of cytokinin screening assay to determine concentration ranges for plaque formation assays and single-step growth curves. EPC cells were concurrently infected with FV3 and treated with exogenous (A) iP or (B) iPR, 0-80µM for 24 hours. After 24 hours, cells were overlaid with 0.75% methylcellulose in L-15 media supplemented with 1% FBS. Plaque formation was assessed 72 hours post-infection. Data are presented as mean plaque reduction relative to the control ± SEM. Statistical significance was evaluated using a Kruskal-Wallis followed by Dunn's post-hoc analysis (n≥3; * $p \leq 0.05$, *** $p \leq 0.001$, ****$p \leq 0.0001$). In GraphPad Prism 8, interpolation analysis (Sigmoidal, 4PL, X is concentration) was used to determine cytokinin concentrations for 75% maximum plaque reduction used in single-step growth curve experiments (highlighted in red; 16.5µM iP, 16µM iPR).
