# Supplementary material for: Cytokinins Reduce Viral Replication and Alter Plaque Morphology of Frog Virus 3 In Vitro
Source: Viruses. 2024 May 23;16(6):826. doi: 10.3390/v16060826 (PMC11209418; doi:10.3390/v16060826)
Supplement: Supplementary file 1 [file viruses-16-00826-s001.zip › Table S1.pdf]

Supplementary Table S1. Cytotoxic and proliferative effect of cytokinins on EPC cells. Cytotoxicity and proliferative effects of all compounds were evaluated using the Cell Counting Kit 8 assay (WST-8/CCK8; Abcam, Toronto, ON, Canada). CK treatments were applied at 30 µM in 1% L-15 at 20-25°C. EPC cells were treated for 24 hours to evaluate cytotoxicity. To evaluate proliferation, after 24h CK exposure supernatants were replaced with fresh 1% L-15 and plates were incubated for an additional 48h. Cytotoxicity (CC50) and proliferative effects (EC50) were determined after absorbance (OD 460 nm) was measured at endpoints. Data are presented as the average of two repeated assays each comprising three replicates.

Compound Name	CC_{50} + SE[a]	EC_{50} + SE[a]
N^6-isopentenyladenine	>30	>30
N^6-isopentenyladenosine	>30	>30
N^6-isopentenyladenosine 5'-monophosphate	>30	>30
2-methylthiol N^6-isopentenyladenosine 5'-monophosphate	>30	>30

[a]All values are in µM, based on 2 independent experiments comprising 3 replicates each.
